# High bleeding rates in δ-storage pool disease during surgeries and deliveries

**DOI:** 10.1016/j.rpth.2025.103239

**Published:** 2025-10-30

**Authors:** Elleke van Heerwaarde, Annick de Moor, Albert Huisman, Rolf Urbanus, Idske Kremer Hovinga, Roger Schutgens, Thrombocytopathy in the Netherlands (TiN) study group

**Affiliations:** 1 Center for Benign Haematology, Thrombosis and Haemostasis, Van Creveldkliniek, Utrecht University, Utrecht, The Netherlands; 2 Central Diagnostic Laboratory, University Medical Center Utrecht, Utrecht University, Utrecht, The Netherlands

**Keywords:** blood platelet transfusion, hemorrhage, perioperative care, platelet storage pool deficiency, postpartum hemorrhage

## Abstract

**Background:**

δ-storage pool disease (δ-SPD) involves various platelet disorders due to deficient or reduced secretion of dense granules, causing increased bleeding tendency, particularly during surgery and delivery. The optimal treatment to prevent bleeding in δ-SPD is unknown.

**Objectives:**

To evaluate the efficacy of preventive treatment in a cohort of patients with δ-SPD.

**Methods:**

In a single-center study, we retrospectively reviewed the medical files of δ-SPD patients diagnosed between 2016 and 2024. Data included invasive diagnostic procedures, surgeries (minor/major), and deliveries (vaginal/cesarean section) before and after diagnosis. Diagnosis was confirmed by platelet adenosine diphosphate levels (<1.4 μmol/10^11^ platelets). Bleeding complications were defined according to the International Society on Thrombosis and Haemostasis bleeding scale. Data on treatment and platelet transfusions were documented.

**Results:**

Thirty-eight patients (mean age, 35.6 years; range, 0-67; 63.2% female) were included. The average adenosine diphosphate level was 0.82 μmol/10^11^ platelets (range, 0.2-1.4). A total of 161 interventions and deliveries were analyzed. The bleeding rate was dependent on the timing of diagnosis (51.5% before δ-SPD diagnosis and 16.8% after; *P* < .001) and on the use of preventive treatment (45.8% without and 17.9% with; *P* < .001). A total of 145 interventions were analyzed, including 63 minor and 82 major. Of these, 43/145 (29.7%) were complicated by bleeding (39 minor bleeds and 4 major bleeds). Sixteen deliveries were analyzed. Among 10 vaginal deliveries, 9 had postpartum hemorrhage, of whom 6 had no prophylaxis. All 6 cesarean sections received prophylactic platelet transfusion; no bleeding occurred.

**Conclusion:**

We observed a high perioperative/peripartum bleeding rate in patients with δ-SPD. This decreased significantly after a correct diagnosis. In addition, bleeding rates were significantly lower among patients who received preventive treatment before the procedure.

## Essentials

• We evaluated the efficacy of preventive treatment in patients with δ-storage pool disease (δ-SPD).
• Medical records of δ-SPD patients were reviewed for procedures and deliveries, as well as bleeding outcomes.
• Bleeding rates were significantly lower after diagnosis and prophylactic treatment.
• Proper diagnosis and preventive measures can reduce bleeding complications in δ-SPD patients.

## 1 Introduction

Storage pool disease (SPD) is a group of inherited platelet disorders caused by a deficiency or reduced secretion of platelet storage granules [1,2]. Platelet granules are small organelles in platelets containing molecules that play an important role in coagulation. Granules release their contents after the activation of the platelets, causing platelet aggregation and further activation [3]. There are 3 types of platelet granules: α-granules, dense (or δ) granules, and lysosomes [3,4]. SPD can occur as syndromic or nonsyndromic SPD. Based on the type of platelet granules involved, syndromic SPD can be categorized as α-SPD (Gray platelet syndrome, Quebec platelet disorder, renal dysfunction, and cholestasis syndrome), δ-SPD (Hermansky–Pudlak syndrome [HPS], Chediak–Higashi syndrome, and Gricelli syndrome), or αδ-SPD (X-linked thrombocytopenia, thalassemia, Jacobsen syndrome, and Wiskott–Aldrich syndrome). SPD can be isolated or combined with other platelet function deficiencies (RUNX1 or GFI1b mutations) [4].

δ-SPD is generally characterized by mild symptoms, such as easy bruising and epistaxis, caused by an increased mucocutaneous bleeding tendency [[1], [2], [3]]. However, during surgical procedures or deliveries, more severe bleeding complications can occur. To prevent patients from bleeding, preventive treatment can be given before surgery. There are currently 3 types of preventive treatment for SPD patients: tranexamic acid (TXA), desmopressin (DDAVP), or platelet transfusion (PT), which can also be given in combination. Because SPD is a rare disorder, little is known about the optimal treatment strategy. The Surgery in Platelet Disorders and Therapeutic Approach study from the Scientific Working Group on Thrombocytopenias and Platelet Function Disorders of the European Hematology Association revealed that excessive bleeding after surgery occurred in 20% of patients with inherited platelet function defects. Details of all patients with δ-SPD were not given, although the bleeding rate was 27% in 22 patients with HPS and 24% in 10 patients with Gray platelet syndrome [5].

In this study, we performed a retrospective analysis of the clinical course of interventions and deliveries in patients with δ-SPD before and after diagnosis.

## 2 Methods

### 2.1 Study population

In this single-center study, a retrospective analysis was performed using clinical records from patients’ medical files. Patients diagnosed with δ-SPD at our center between January 2016 and May 2024 were included. This study was approved by the Medical Ethics Review Committee, and informed consent was obtained from all participants.

Data were gathered regarding the characteristics of the patients, genetic diagnostics for causative DNA mutations, and all medical interventions related to invasive diagnostic procedures, surgeries, and deliveries. Clinical data included both interventions and deliveries performed at the University Medical Center (UMC) Utrecht, as well as historical information about prior interventions and deliveries. Historical data were retrieved from patients' medical records, either through anamnestically reported information during clinical consultations or through correspondence from other hospitals and general practitioners. All lifetime interventions and deliveries were documented, including those conducted prior to diagnosis. Interventions and deliveries were excluded when data on the type of preventive treatment or bleeding outcome were incomplete.

δ-SPD was diagnosed based on the platelet adenosine diphosphate (ADP) content as described previously [6]. ADP and adenosine triphosphate (ATP) platelet content were measured in accordance with the ISO 15189-certified Central Diagnostic Laboratory of the UMC Utrecht. In short, ADP was converted to ATP and subsequently measured using the ATPLite 1-Step Kit (PerkinElmer) on a Synergy HTX luminometer (Agilent) according to the manufacturer's protocol. ADP levels were expressed in μmol/10^11^ platelets. The ADP and ATP reference interval was determined using 40 healthy donors. δ-SPD was determined if a patient had at least 2 times a platelet ADP content below the reference value of 1.4 μmol/10^11^ platelets. For analysis, the lowest ADP level measured was used.

Gene variants associated with platelet disorders were analyzed by whole-exome sequencing using a targeted gene panel for primary hemostasis, as described previously [7].

### 2.2 Classifications and definitions

Surgeries were classified as “major” or “minor” according to the classification system described by Solimeno et al. [8] for people with hemophiliaKlik of tik om tekst in te voeren. Since the article by Solimeno et al. [8] was a literature review, certain interventions were recorded multiple times and may have been classified as either minor or major in different articles. In such cases, the most common classification was used in this analysis. For the classification of bleeding events after intervention, the International Society on Thrombosis and Haemostasis (ISTH) bleeding scale was used [9]. Nonmajor bleedings were classified as minor bleeding. Deliveries were subdivided into vaginal deliveries and cesarean sections. Bleeding events after delivery were classified as postpartum hemorrhage (PPH) when blood loss was more than 500 mL according to World Health Organization guidelines [10].

### 2.3 Statistical analysis

The chi-square test was used to compare categorical data in patients undergoing interventions and deliveries. SPSS Statistics 30.0.0 (IBM Corporation) was used for all analyses, with a significance level set at *P* < .05.

## 3 Results

### 3.1 Patient characteristics

A total of 38 patients were included; see Table 1 for the characteristics. Genetic data were available for 23 patients, and causative mutations were identified in 10 of them.

**Table 1:** Patient characteristics.

Characteristic	*N* (%), count (%) or mean (range)
Total patients, *N*	38
Females, *n* (%)	24 (63.2)
Males, *n* (%)	14 (36.8)
Age (y), mean (range)	35.6 (0-67)
Platelet ADP and ATP content (μmol/10^11^ platelets), mean (range)	
ADP (1.4-3.3)	0.82 (0.2-1.4)
ATP (2.7-4.8)	3.2 (2.2-4.4)
ATP/ADP ratio (1.20-2.00)	4.72 (2.16-15.65)
Platelet count × 10^9^/L (150-450), mean (range)	212 (52-421)
Platelet count < 150 × 10^9^/L, *n* (%)	11 (28.9)
Platelet count < 100 × 10^9^/L, *n* (%)	6 (15.8)
Causative genetic mutations, *n*	
RUNX1	5
Deletion of chromosome 11 (11qter)	2
RBM8A	1
GATA1	1
GFI1B	1

ADP, adenosine diphosphate; ATP, adenosine triphosphate.

### 3.2 Bleeding outcomes

In total, 161 procedures and deliveries were included in the study (Table 2). Bleeding occurred in 38 of 83 cases (45.8%) without preventive treatment compared with 14 of 78 cases (17.9%) with preventive treatment (*P* < .001).

**Table 2:** Preventive treatment according to the type of intervention and bleeding events.

Minor interventions	Total *n* = 63	Minor bleeding event	Major bleeding event	Total bleeds
Without preventive treatment	38 (60.3)	10 (26.3)	0	10 (26.3)
With preventive treatment				
TXA only	9	0	0	
DDAVP only	4	1	0	
TXA + DDAVP	4	0	0	
PT	7	1	0	
TXA + DDAVP + PT	1	0	0	
Total	25 (39.7)	2 (8.0)		2 (8.0)

Major interventions	Total *n* = 82	Minor bleeding event	Major bleeding event	Total bleeds
Without preventive treatment	39 (47.6)	19 (48.7)	3 (7.7)	22 (56.4)
With preventive treatment				
DDAVP only	7	4	0	4
TXA + DDAVP	9	2	0	2
PT	23	1	1	2
TXA + PT	2	0	0	0
DDAVP + PT	1	1	0	1
TXA + DDAVP + PT	1	0	0	0
Total	43 (52.4)	8 (18.6)	1 (2.3)	9 (20.9)

Vaginal deliveries	Total *n* = 10	PPH		
Without preventive treatment	6	6 (100)		
TXA + DDAVP	4	3 (75)		

Cesarean sections	Total *n* = 6	PPH		
PT	6	0		

Data are given as numbers or *n* (%).

DDAVP, desmopressin; PPH, postpartum hemorrhage; PT, platelet transfusion; TXA, tranexamic acid.

Tables 3 and 4 present the distribution of bleeding events associated with interventions and deliveries, categorized by whether they occurred before or after the diagnosis of δ-SPD and whether preventive treatment was administered. Before the diagnosis of δ-SPD, 35 of 68 interventions and deliveries (51.5%) were associated with bleeding events, and after the diagnosis, 17 of 93 interventions and deliveries (18.3%) were associated with bleeding events (*P* < .001). Of 83 untreated procedures, 62 occurred prior to the diagnosis of δ-SPD (74.7%).

**Table 3:** Distribution of bleeding events and interventions by diagnosis status and treatment.

Treatment group	Total *n*	No bleeding *n* (%)	Minor bleeding *n* (%)	Major bleeding *n* (%)	Total bleeds *n* (%)
**Before diagnosis**					
Without preventive treatment	57	30 (52.6)	25 (43.9)	2 (3.5)	27 (47.4)
With preventive treatment^a^	4	1 (25)	3 (75)	0	3 (75)
**After diagnosis**					
Without preventive treatment	20	15 (75)	4 (20)	1 (5)	5 (25)
With preventive treatment^a^	64	56 (87.5)	7 (10.9)	1 (1.6)	8 (12.5)

^a^Preventive treatment includes tranexamic acid, desmopressin, platelet transfusion, or a combination of these.

**Table 4:** Distribution of postpartum hemorrhage and deliveries by diagnosis status and treatment.

Treatment	Total *n*	No bleeding *n* (%)	PPH *n* (%)
**Before diagnosis**			
Without preventive treatment	5	0	5 (100)
With preventive treatment^a^	2	2 (100)	0
**After diagnosis**			
Without preventive treatment	1	0	1 (100)
With preventive treatment^a^	8	5 (62.5)	3 (37.5)

PPH, postpartum hemorrhage.

a Preventive treatment includes tranexamic acid, desmopressin, platelet transfusion, or a combination of these.

#### 3.2.1 Interventions

A total of 145 interventions were recorded, consisting of 63 minor and 82 major procedures. A total of 43 cases (29.7%) were associated with bleeding complications. Of these, 39 were classified as minor bleeding events and 4 as major bleeding events (Table 2).

Among the minor interventions, the most frequent procedures were minor dental procedures (30.2%). The minor dental procedures category included 17 dental extractions (excluding wisdom tooth extractions), 1 apicoectomy, and 1 dental hygiene procedure. Genital procedures consisted of 1 circumcision and 1 intrauterine device insertion. Among the major interventions, the most commonly reported procedures were major dental interventions (32.9%), abdominal surgeries (17.1%), and orthopedic surgeries (14.6%). The major dental procedures category included 1 case involving 3 simultaneous tooth extractions, while the remaining cases were wisdom tooth extractions (Supplementary Table).

For procedures performed at UMC Utrecht, DDAVP was administered as a single prophylactic dose of 0.3 μg/kg. Data on DDAVP use were not available for procedures performed at other centers. TXA prophylaxis at UMC Utrecht consisted of 1500 mg 3 times daily for 5 to 7 days. In other hospitals, TXA was given according to local protocols, but detailed information about these centers was not available.

In our cohort, 6 patients had a platelet count below 100,000/μL (Table 1). These patients underwent a total of 24 procedures, of which PT was administered in 5 cases. No additional doses were given.

#### 3.2.2 Minor interventions

In total, 19.0% of patients experienced a bleeding event following a minor intervention (Table 2). All bleeding events were classified as minor bleeding. The majority of minor interventions (60.3%) were performed without any preventive treatment. The incidence of bleeding complications was 26.3% in patients who did not receive preventive treatment compared with 8.0% in those who received preventive treatment (*P* = .07). TXA alone was given in 9 cases: 3 injection procedures, 2 dental extractions, 1 endoscopic procedure, 1 intrauterine device insertion, 1 soft tissue excision, and 1 urological procedure. In this group, no bleeding events were observed. The procedures in which DDAVP was used as a preventive treatment included 2 puncture procedures, 1 soft tissue excision, and 1 cardiovascular device implantation. Only soft tissue excision resulted in bleeding. In the 4 cases where both TXA and DDAVP were used in combination, comprising 2 endoscopic procedures, 1 dental extraction, and 1 apicoectomy, no bleeds were reported. PT was administered in 7 cases: 2 injection procedures (1 unit of platelets), 1 biopsy (2 units of platelets), 1 cardiovascular device implantation (the number of transfusions was unrecorded), 1 puncture procedure (1 unit of platelets), 1 endoscopic procedure (1 unit of platelets), and 1 circumcision (1 unit of platelets). Only the circumcision resulted in a minor bleeding event postsurgery. A combination of TXA, DDAVP, and PT was administered for a procedure that included a biopsy and urological procedure; no bleeding occurred.

#### 3.2.3 Major interventions

In total, 37.8% of patients who underwent a major intervention reported a bleeding event, with 27 events classified as minor bleeding and 4 as major bleeding. Most patients (52.4%) received some form of preventive treatment, while no preventive treatment was given in 39 cases (61.9%). The incidence of bleeding complications was higher among patients who did not receive preventive treatment (56.4%) than among those who received prophylaxis (20.9%; *P* < .001). High bleeding rates were observed after wisdom tooth extractions, occurring in 66.7% of cases without prophylaxis and in 20% with prophylaxis. Other bleeding events were associated with various surgeries, including tonsillectomy, neurological, abdominal, and gynecological procedures. In the group that did not receive prophylaxis, 3 bleeding events were classified as major. One required reoperation following orthopedic surgery, 1 involved a tonsillectomy that required reoperation with suturing, and the other involved a decrease in hemoglobin level of >2 g/dL after cardiothoracic surgery. DDAVP was administered in 7 procedures, including 2 wisdom tooth extractions, 2 abdominal surgeries, 1 thyroid surgery, 1 orthopedic surgery, and 1 gynecological surgery. Bleeding occurred in 4 cases: 1 thyroid surgery, 1 wisdom tooth extraction, 1 abdominal surgery, and 1 gynecological surgery. A combination of TXA and DDAVP was used in 9 cases, consisting of 8 wisdom tooth extractions and 1 dental procedure involving 3 simultaneous tooth extractions. Two of the wisdom tooth extractions resulted in bleeding. PT was administered prior to 23 procedures, including 6 abdominal surgeries, 5 orthopedic surgeries, 4 neurological surgeries, 3 wisdom tooth extractions, 2 gynecological surgeries, 1 plastic surgery, 1 urological surgery, and 1 vascular surgery. Postoperative bleeding occurred only after the urological surgery. It was classified as a major bleeding event because a second endovascular procedure was necessary to stop the bleeding. The combination of TXA and PT was given in 2 cases, specifically a wisdom tooth extraction and a urological surgery, with no subsequent bleeding. A combination of DDAVP and PT was administered for a neurological surgery, which resulted in minor bleeding. A combination of TXA, DDAVP, and PT was used in 1 neurological surgery, with no postoperative bleeding observed.

#### 3.2.4 Deliveries

A total of 16 deliveries were reported, including 10 vaginal deliveries and 6 cesarean sections (Table 2). The prevalence of PPH was 90% in 10 vaginal deliveries. Among the 6 patients who did not receive preventive treatment, 6 of 6 (100%) experienced PPH, and among the 4 patients who received TXA and DDAVP as prophylaxis, 3 of 4 (75%) experienced PPH (*P* = .197). All 6 cases of cesarean sections received prophylactic treatment, and no cases of PPH were reported.

## 4 Discussion

In this study, 161 interventions and deliveries of 38 patients with a δ-SPD diagnosis were investigated for bleeding complications, demonstrating that the bleeding rate was dependent on the timing of diagnosis (51.5% had bleeding complications before δ-SPD diagnosis and 18.3% after; *P* < .001) and the use of preventive treatment therapy (45.8% without and 17.9% with; *P* < .001). Additionally, in deliveries without prophylaxis, the incidence of PPH was notably high. These findings suggest that early diagnosis and prophylactic treatment help reduce bleeding risks.

Our data demonstrated a higher bleeding rate than expected in δ-SPD patients. Overall, more than a quarter of the minor procedures were complicated by bleeding events, especially when no preventive measures were taken. Notably, one surgery performed without prophylaxis resulted in a major bleeding event. In 25% of patients receiving DDAVP, bleeding occurred, and in 1 surgery using PT as prophylaxis, a bleeding event still occurred. These bleeding events may be attributed to the more invasive nature of these interventions. Additionally, differences in phenotype among patients with SPD could explain variability in bleeding risk. The majority of patients who underwent a major intervention after diagnosis received preventive treatment, especially PT. PT was effective in 21 of 23 interventions, whereas more than half of the patients who did not receive any form of treatment experienced bleeding. This emphasizes the importance of discussing preventive treatment in SPD patients undergoing surgery.

It is noteworthy that causal variants were identified in 10 out of 23 sequenced patients, most frequently involving RUNX1, suggesting that genetic testing can provide potentially prognostic information to individuals with δ-SPD.

It is important to note that in our study, we used an existing classification system to stratify the risk of intervention [8]. We would like to highlight 2 specific points regarding this system. First, because no system exists that stratifies procedural bleeding risk specifically for platelet disorders, we applied the hemophilia-based framework. While platelet disorders typically manifest with mucocutaneous bleeding, this does not imply a higher risk of procedure-related mucosal bleeding than in hemophilia. Therefore, we consider this approach to be the most appropriate and pragmatic available option. Second, we deviated from the hemophilia-based classification for tonsillectomy. Bleeding is a significant complication of tonsillectomies, even in patients without bleeding disorders. Although this procedure is classified as minor in the hemophilia framework, we considered it a major intervention in our study.

Bleeding complications in δ-SPD have been investigated previously. A study investigated bleeding during surgery in patients with inherited platelet disorders. They demonstrated that the bleeding rate in minor and major interventions without preventive therapy was 21.4% and 20.8%, respectively, and in dental interventions, it was 16.7%, which is lower than in our study (26.3% for minor interventions, 56.4% for major interventions, 66.7% for wisdom tooth extractions, and 33.3% for minor dental interventions). It is important to note, however, that they applied their own post hoc criteria to classify interventions as minor or major, which differ from the classification used in our study. This may affect the reliability of the comparison. Their study included 54 patients with a type of δ-SPD (17 δ-SPD patients, 22 HPS, 13 familial platelet disorders and predisposition to acute myelogenous leukemia [RUNX1], and 2 combined αδ-granule deficiency). However, in this study, patients received prophylaxis more often. Detailed data on SPD patients could not be retrieved from the dataset; therefore, the effect of prophylaxis on the surgical bleeding rate in the SPD group could not be determined [5].

Although most bleeding events in this study were minor according to the ISTH bleeding scale, many still required treatment or (prolonged) hospital admission. Thus, the impact on the patient is still important.

We observed an alarmingly high rate of PPH in our patients. Clearly, this indicates that vaginal deliveries without preventive treatment are inadequate. Our data suggest that prophylaxis with DDAVP and TXA may be insufficient for patients with SPD to prevent PPH. However, due to the small sample size, no firm conclusions can be drawn. Nevertheless, these observations highlight the importance of proactive management in this setting. In all 6 cases of cesarean sections, PT seems to prevent bleeding. It should be noted that these 6 procedures were performed in only 2 patients. The study by Civaschi et al. [11] investigated PPH in 5 different platelet disorders, including SPD. They found bleeding complications in 13.9% of 40 deliveries in patients with SPD, which is lower than in our study (56.3% of 16 deliveries). In 5 cases, PT was given as preventive treatment, and no bleeding complications were observed in this group, consistent with our results [11]. Although the results do not completely correspond, both studies show that preventive treatment protects against bleeding. Therefore, attention should be given to the peripartum management of SPD patients.

This study has several limitations. First, due to the retrospective nature of the data collection, some data were missing. Information about the type of preventive treatment and bleeding events was not regularly reported, so we could not include all procedures. Some procedures were performed before diagnosis; therefore, the information was not specifically documented. Second, some interventions were relatively scarce. Therefore, subgroup analysis could not be performed. Third, some patients had platelet function disorders in addition to δ-SPD, ie, thrombocytopenia and/or diminished luminoaggregometry in RUNX1 patients and an α-granule defect in Jacobsen syndrome. However, none of the patients had platelet counts below 50 × 10e9/L, and the bleeding rates in this group were consistent with the overall results of the study.

The main strength of this study is its relatively large cohort of patients. SPD is a rare disease, so it is exceptional to have a cohort of 38 patients within a single center. Additionally, this cohort underwent a large number of interventions, allowing us to compare different types of treatment across both minor and major interventions.

This study demonstrates that bleeding complications during interventions in patients with δ-SPD are not uncommon, particularly prior to diagnosis. Therefore, prophylactic treatment should be considered for all types of interventions, especially major surgeries and deliveries. PT should be considered for cesarean sections and major surgery, whereas minor interventions can often be treated with DDAVP in combination with TXA. PT may be preferred over DDAVP for adenotonsillectomy.
