# Supplementary material for: High bleeding rates in δ-storage pool disease during surgeries and deliveries
Source: Res Pract Thromb Haemost. 2025 Oct 30;9(8):103239. doi: 10.1016/j.rpth.2025.103239 (PMC12681540; doi:10.1016/j.rpth.2025.103239)
Supplement: Supplementary Table 1 [file mmc1.docx]

SupplementaryTable 1. Overview of 145 interventions in 38 δ-SPD patients

| **Procedure** | **N (%)** |
| --- | --- |
| **MINOR INTERVENTIONS (n=63)** |  |
| Dental procedures | 19 (30.2) |
| Soft tissue excision/incision | 9 (14.3) |
| Endoscopic procedures | 9 (14.3) |
| Puncture/injection procedures | 9 (14.3) |
| Urological procedures | 5 (7.9) |
| Biopsy | 2 (3.2) |
| Genital procedures | 2 (3.2) |
| Cardiovascular device implantation | 2 (3.2) |
| Finger surgery procedures | 2 (3.2) |
| Hernia repair | 1 (1.6) |
| Ophtalmic procedures | 1 (1.6) |
| Cardiac catheterization | 1 (1.6) |
| Adenoidectomy | 1 (1.6) |
| **MAJOR INTERVENTIONS (n=82)** |  |
| Dental procedures | 27 (32.9) |
| Abdominal surgery | 14 (17.1) |
| Orthopedic surgery | 12 (14.6) |
| Tonsillectomy | 10 (12.2) |
| Gynecological surgery | 6 (7.3) |
| Neurosurgery | 6 (7.3) |
| Nasal surgery | 2 (2.4) |
| Cardiothoracic surgery | 1 (1.2) |
| Vascular surgery | 1 (1.2) |
| Plastic & reconstructive surgery | 1 (1.2) |
| Urological surgery | 1 (1.2) |
| Thyroid surgery | 1 (1.2) |
